# Phase Angle, Inflammation, and Sarcopenia in Late Postoperative Roux-En-Y Gastric Bypass

**DOI:** 10.3390/jcm12155124

**Published:** 2023-08-04

**Authors:** Gisele Florêncio, Aglécio Souza, Elinton Chaim, Allan Santos, Louise Duran, Camila Carvalho, Sarah Monte Alegre

**Affiliations:** 1 Department of Internal Medicine, Faculty of Medical Sciences, State University of Campinas (UNICAMP), Campinas 13083-887, Brazil; 2 Department of Surgery, Faculty of Medical Sciences, State University of Campinas (UNICAMP), Campinas 13083-887, Brazil; 3 Department of Nuclear Medicine, Clinic Hospital, State University of Campinas (UNICAMP), Campinas 13083-887, Brazil

**Keywords:** phase angle, sarcopenia, sarcopenic obesity, obesity, Roux-en-Y bypass gastric

## Abstract

Sarcopenic obesity is characterized by a disproportion between the amount of muscle to fat. Contrary to most studies evaluating parameters related to sarcopenic obesity in the elderly, this study aims to evaluate the phase angle (PhA) and sarcopenia in young individuals pre- and post-Roux-en-Y gastric bypass. A total of 69 volunteers (46 women and 23 men; 38.5 ± 8.1 years) participated in this study. Body composition and PhA were assessed using BIA. Sarcopenia was assessed using a handgrip strength test (HGS) and gait speed (GS), and appendicular lean mass (ALM) was assessed using Dual Energy X-ray Absorptiometry (DXA). The PhA was significantly lower (*p* < 0.0007) and the resistance (R) significantly higher (*p* = 0.0026) in the postoperative group. HGS was negatively correlated with R (r = −0.63669; *p* < 0.0001), hs-CRP (r = −0.45436; *p* = 0.0197), and leptin (r = −0.46505; *p* = 0.0043). GS was negatively correlated with R (r = −0.36220; *p* = 0.0254), and ALM was negatively correlated with reactance (r = −0.49485; *p* = 0.0034) and R (r = −0.65797; *p* ≤ 0.0001). PhA and other components of BIA provide a good correlation with sarcopenia, especially regarding the reduction in muscle function, in an early form, in individuals in the pre- and postoperative period of gastric bypass.

## 1. Introduction

The term sarcopenic obesity refers to the presence of sarcopenia and obesity [1]. Such a condition shows a disproportion between the amount of muscle to fat, with a smaller amount of muscle [2]. Sarcopenia is a widespread progressive loss of strength and muscle mass, associated with age and more prevalent in the elderly, leading to functional limitations, increased comorbidities, and mortality [3]. Possible mechanisms for the loss of muscle quality, mass, and strength include capillary density and reduced innervation strength of skeletal muscle, in addition to atrophy of type II muscle fibers [4,5]. Sarcopenia progression involves other mechanisms such as genetic factors, neuromuscular integrity, hormonal factors, oxidative stress, physical inactivity, inadequate diet, and increased inflammatory process [6].

The phenotypic differences in body composition between obesity patients with and without sarcopenia are individuals with obesity having high body weight, fat mass (FM), body mass index (BMI), waist circumference (WC), and normal or high appendicular lean mass (ALM); in contrast, those with obesity and sarcopenia have body weight, fat mass, BMI, and WC from normal to high and have low ALM [7].

Among the most accurate methods of assessing body composition are magnetic resonance imaging (MRI), computed tomography (CT), and Dual Energy X-ray Absorptiometry (DXA) [8,9], but they are considered invasive and costly methods, not being eligible for use in clinical practice [10]. Analysis using electrical bioimpedance (BIA) has been reported as a validated, low-cost method of evaluating body composition and is applicable in clinical practice. Its accuracy has been validated with DXA and CT in different populations [11,12]. It provides estimated fat mass and muscle mass values through predictive equations and anthropometric parameters [12].

The phase angle (PhA) is a variable derived from reactance (Xc) and resistance (R), indexes available in BIA. It is essential for assessing cell membrane integrity [13]; it is also associated with inflammatory status and some diseases, including obesity [14] and, more recently, sarcopenia [15,16,17]. Most studies evaluate parameters related to sarcopenia and sarcopenic obesity in the elderly. This study aimed to assess the phase angle and sarcopenia in young individuals in the preoperative and late postoperative periods of gastric bypass by Roux-en-Y.

## 2. Materials and Methods

### 2.1. Design and Settings

This work is a cross-sectional study at the Metabolic Unit of the Department of Clinical Medicine, Faculty of Medical Sciences-UNICAMP, (Campinas, SP, Brazil). The Research Ethics Committee of the Faculty of Medical Sciences-UNICAMP approved this project (opinion number 1708683/2016). Before the interview, to collect data on the volunteers’ clinical, personal, and family histories, the research aims were clarified, and subjects signed the terms of the Free and Informed Consent Form (ICF).

### 2.2. Subjects

The subjects were men and women aged 18–59 years; all sedentary (less than 150 min per week of physical activity) [18]; BMI ≥ 35 kg/m^2^ with comorbidities or ≥40 kg/m^2^; a group not submitted to bariatric surgical procedures; a group submitted to gastric bypass surgery 2–5 years ago at the Bariatric Surgery Outpatient Clinic (Hospital de Clínicas-UNICAMP). Exclusion criteria: subjects with heart disease, uncontrolled Systemic Arterial Hypertension (SAH) (≥160/100 mmHg), physically active (≥150 min per week), those with malignant diseases, kidney disease, chronic liver disease, hyper- or hypothyroidism, in treatment with statins, using insulin, contraceptives, in addition to pregnant or lactating women and menopausal women.

**Figure 1:**
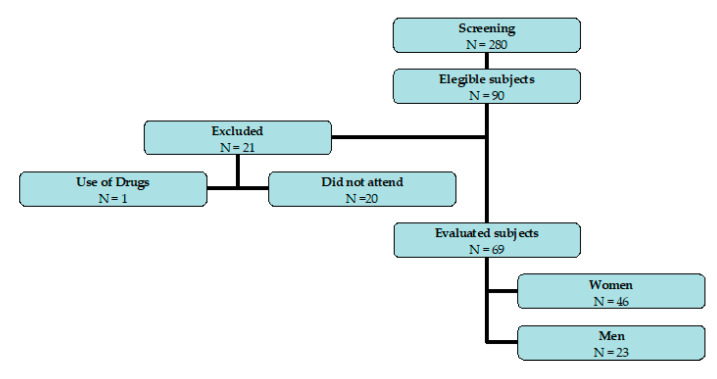
Flow chart of the study.

The researchers screened 280 volunteers, initially selecting 90, excluding 20 for not attending the exams and 1 for using a contraceptive medication, ending the study with 69 volunteers (46 women (66.7%) and 23 men (33.3%) (Figure 1)).

### 2.3. Anthropometric Measurements

After 10–12 h of fasting, the volunteers came to the Metabolic Unit of the Hospital de Clinicas, UNICAMP at 7:30 a.m., wearing light clothes to measure anthropometric parameters—weight in kilograms (kg), height in meters (m), waist circumference (WC), and neck circumference (NC) in centimeters (cm)—by a single evaluator and perform the exams. The above parameters were evaluated as follows: body mass on the Welmy brand mechanical scale with 100 g precision and a 200 kg capacity; height using a stadiometer attached to the scale and accurate to 0.5 cm; WC with a flexible inelastic measuring tape 2 m in length; all measurements performed as previously described by [19]; NC measured at the height of the midpoint of the neck, or just below the prominence in men with a prominent larynx (Adam’s apple) [20]; BMI used to assess nutritional status (BMI = weight (kg)/height(m)²) and classified according to [21,22].

### 2.4. Body Composition Analysis

The BIA equipment Biodynamics Bioimpedance Analyzer model 310 was used to assess the body compartment FM (kg), fat-free mass (kg) (FFM), % body fat (% BF), and basal metabolic rate (BMR). To perform the test, the volunteers should wear light clothes and not use any metal objects that could interfere with the measurement of the Xc (Ohm) and R (Ohm) vectors. The guidelines of the European Society of Parenteral and Enteral Nutrition (ESPEN) provided a base for the recommendations for carrying out the BIA [23]. The volunteer remained at rest in the lying position for 10 min; the area was properly cleaned with alcohol, and the electrodes (TBW brand, specific for use in BIA models 310 and 450) were positioned on the right side of the body, as previously described by [24]. The PhA (degree) was calculated using the formula: PhA = arctangent Xc/R ((Xc/R) × (180/π)).

### 2.5. Handgrip Strength Assessment (HGS)

HGS was performed using a Crown hydraulic manual dynamometer (50 kgf capacity), according to the protocol recommended by the American Association of Hand Therapists [25].

### 2.6. Gait Speed Assessment

The participant initially stood behind a starting line marked with tape, and, after the voice command “Go!”, started to walk at his usual pace along a 6 m course and stopped after the finish line. The stopwatch was started after crossing the starting line and stopped after crossing the finish line. The measurements were performed in duplicate, with the fastest value in meters/second considered [26].

### 2.7. Appendicular Lean Mass Assessment (ALM)

For the ALM measurement, the Lunar brand equipment model DPX (Lunar Radiation Corporation, Madison, WI, USA) performed the DXA. The patient was positioned in the scanning area of the equipment so that the sagittal line demarcated in this area passed under the center of some anatomical points, such as the skull, spine, pelvis, and legs. ALM corresponds to the lean mass of arms and legs and is defined by the difference between total lean mass and lean change, after disregarding bone content, as defined by [27]. For this study, we only consider the ALM measurements performed by DXA.

### 2.8. Sarcopenia Classification

The criteria for sarcopenia classification are based on the European Working Group on Sarcopenia in Older People (EWGSOP) [6]. ALM < 7.26 kg/m^2^ for men and < 5.45 kg/m^2^ for women; HGS < 30 kgf for men and <20 kgf for women and/or walking speed < 0.8 m/s.

### 2.9. Assay Methods

The subject’s blood samples were collected after 10–12 h of fasting then centrifuged, and the serum was immediately stored in small aliquots in a freezer at −20 °C. The results of insulin and plasma glucose were used in the following formula to calculate the Homeostasis Model Assessment for Insulin Resistance (HOMA-IR): HOMA-IR = Glucose (mg)/18 × Insulin (mUI/L)/22.5 [28]. Values <2.7 were considered normal [29].

Duplicate insulin measurements were performed in serum with an immunoenzymatic assay (ELISA method) using commercial kits of high sensitivity and specificity. Insulin: immunoenzymatic method, ELISA kit (MILLIPORE- Billerica, USA). Sensitivity: 1 µU/mL. Glucose: Enzymatic method, automated using YSI 2300 glucose bioanalyzer equipment. Total cholesterol, Low-Density Lipoprotein cholesterol (LDL-c), High-Density Lipoprotein cholesterol (HDL-c), Very Low-Density Lipoprotein cholesterol (VLDL-c), and Triglycerides (TG): automated colorimetric enzyme method, Roche Diagnostics, on Hitachi 917 equipment (Roche Diagnostics, Mannheim, Germany). Leptin: enzimatic method, ELISA kit (MILLIPORE- Billerica, MI, USA). Sensitivity: 0.195 ng/mL. hs-CRP: immunonephelometry using the nephelometer method, BN II Systems (Siemens DadeBehring Inc., Newark, DE, EUA). Sensitivity: 0.02 mg/dL.

### 2.10. Statistical Analysis

The results were presented as mean and standard deviation (SD). Categorical variables were compared using the Chi-squared test and, when necessary, Fisher’s exact test. For the numerical variables, the Mann–Whitney test was used. Spearman’s linear correlation coefficient was used to assess the relationship between the parameters and the sarcopenia variables. The level of significance adopted was 5%. Data were analyzed using the SAS System for Windows (Statistical Analyzes System), version 9.4.

## 3. Results

A total of 69 volunteers (46 women (66.7%) and 23 men (33.3%)) were evaluated between November 2016 and December 2018. Participants were divided into two groups: (I) preoperative gastric bypass group (n = 39) and (II) postoperative gastric bypass group (n = 30). Table 1 shows characteristics in pre- and postoperative groups stratified by sex. In the preoperative group, there were 28 women and 11 men, and in the postoperative group, there were 18 women and 12 men. We found significant differences between the groups regarding anthropometric parameters weight, BMI, WC, and NC (*p* < 0.0001) for men and women.

Table 2 describes body composition and bioimpedance parameters in pre- and postoperative groups. Concerning body composition, the preoperative group showed a higher fat mass, fat-free mass, and % body fat (*p* < 0.0001) when compared to the postoperative group. The phase angle was significantly smaller (*p* < 0.0007) and the resistance was significantly higher (*p* = 0.0026) in the postoperative group.

Table 3 shows the number of participants in each group classified with sarcopenia according to the criteria established by [6].

Table 4 describes biochemical, metabolic, and inflammatory parameters in preoperative and postoperative groups. As described, the biochemical parameters insulin, glucose, uric acid, gamma-glutamyl transpeptidase (GGT), total cholesterol, LDL-c, VLDL-c, and triglycerides (*p* < 0.0001), in addition to glycated hemoglobin (Hbgli) (*p* = 0.0003) and alanine aminotransferase (ALT) (*p* = 0.0007) were significantly higher and HDL-c was significantly lower (*p* < 0.0001) in the preoperative group. HOMA-IR was higher in the preoperative group when compared to the postoperative group (*p* < 0.0001). The inflammatory parameters hs-CRP and leptin were significantly higher in the preoperative group (*p* < 0.0001).

The correlations between sarcopenia and parameters of BIA, inflammation, and HOMA-IR in the preoperative and postoperative groups are described in Table 5. In the preoperative group, handgrip strength was negatively correlated with resistance (r = −0.63669; *p* < 0.0001), hs-CRP (r = −0.45436; *p* = 0.0197), and leptin (r = −0.46505; *p* = 0.0043), gait speed was negatively correlated with resistance (r = −0.36220; *p* = 0.0254), and ALM was negatively correlated with reactance (r = −0.49485; *p* = 0.0034) and resistance (r = −0.65797; *p* = < 0.0001).

**Figure 2:**
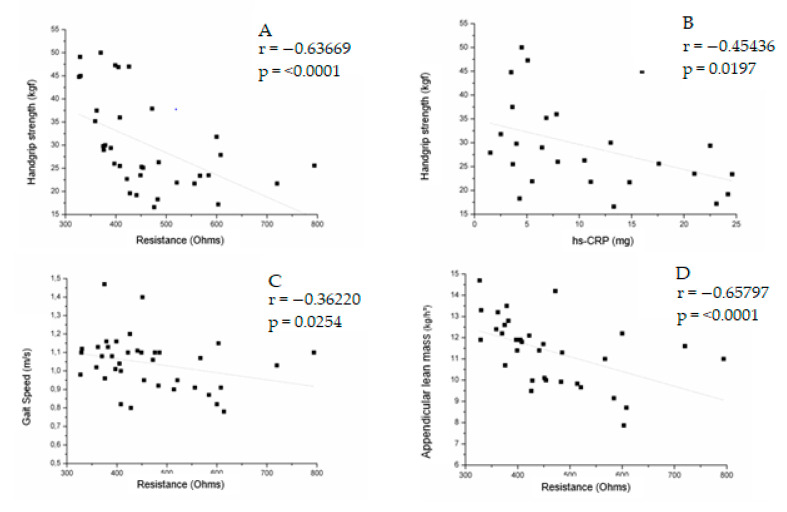

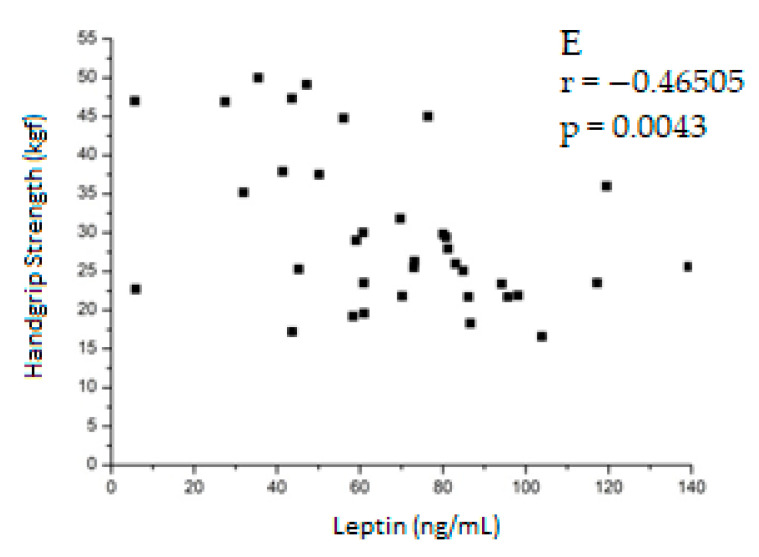
Correlation between handgrip strength and resistance (**A**), handgrip strength and hs-CRP (**B**), gait speed and resistance (**C**), appendicular lean mass and resistance (**D**), handgrip strength and leptin (**E**) in the preoperative group.

In men, gait speed was negatively correlated with HOMA-IR (r = −0.609; *p* = 0.0467) and positively correlated with adiponectin (r = 0.66364; *p* = 0.0260). Handgrip strength was positively correlated with IL1-β (r = 0.83636; *p* = 0.0013), and ALM was negatively correlated with adiponectin (r = −0.68793; *p* = 0.0193). In women, ALM was negatively correlated with reactance (r = −0.64009; *p* = 0.0013) and resistance (r = −0.56538; *p* = 0.0006) (Appendix A Table A1). Figure 2 summarizes the correlations in the preoperative group.

In the postoperative group, handgrip strength was negatively correlated with resistance (r = −0.47147; *p* = 0.0085) and leptin (r = −0.40200; *p* = 0.0277). ALM negatively correlated with resistance (r = −0.72668; *p* < 0.0001) and leptin (r = −0.41000; *p* = 0.0418).

**Figure 3:**
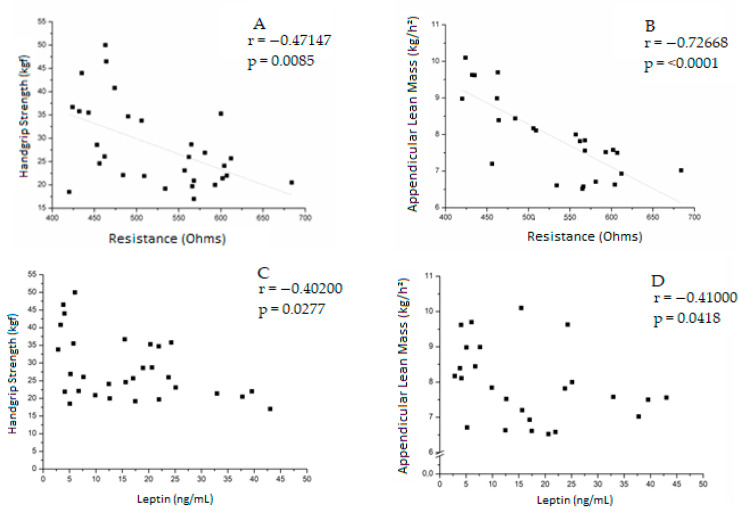
Correlation between handgrip strength and resistance (**A**), appendicular lean mass and resistance (**B**), handgrip strength and leptin (**C**), appendicular lean mass and leptin (**D**) in the postoperative group.

In men, ALM was negatively correlated with resistance (r = −0.788571; *p* = 0.0362). Handgrip strength was positively correlated with adiponectin (r = 0.72727; *p* = 0.0074). (Appendix A Table A2). Figure 3 summarizes the correlations in the postoperative group.

Table 6 compares the classificatory parameters of sarcopenia between the preoperative and postoperative groups of only sarcopenic individuals. The preoperative group presented ALM significantly higher when compared to the postoperative group (*p* = 0.0367).

## 4. Discussion

The evaluation of sarcopenia in patients undergoing gastric bypass is still scarce. The literature data evaluate patients after a recent postoperative period (12 or 18 months) [30,31,32], with accentuated weight loss due to changes in intestinal physiology after a surgical procedure [33] and associated with nutritional deficiencies in 30 to 70% of patients [34], such factors that can be considered bias in body composition assessments.

BIA has been widely used to assess body compartments in clinical practice, and their vectors are correlated with sarcopenia [15,16,17]. We observed significant changes in the resistance and phase angle components in young patients in preoperative and late postoperative gastric bypass. We noted that the phase angle values are lower than the literature’s reference values [35], 4.4° in the preoperative group and 3.7° in the postoperative group. The research also describes that the phase angle reduces with age regardless of body composition [36]. Gómez-Martínez et al. showed PhA < 5.1° in men and <4.8° in women was independently associated with mortality in patients with Chronic Obstructive Pulmonary Disease Patients (COPD) [37]. Low phase angle in COPD patients (<4.5°) was also associated with lower quadricep strength and quality of life [38]. However, our findings indicate that despite being young, they already show significant changes in cell membrane integrity in the preoperative period, being reduced after losing substantial weight by Roux-en-Y bypass (55.27% weight loss in women and 58.93% in men).

Our study shows that 17.86% of women and 9.09% of men in the preoperative period and 22.22% of women and 16.66% of men in the postoperative period present low handgrip strength regarding sarcopenia. As a primary parameter for diagnosing sarcopenia in clinical practice, muscle strength has recently been described as the most reliable muscle function detection indicator. Sarcopenia is confirmed by associating low muscle strength with reduced muscle quantity or quality [39]. In our study, handgrip strength was also negatively correlated with resistance in both groups, and lean appendicular mass negatively correlated with resistance in the postoperative group, indicating body composition’s influence on muscle function and quantity.

The preoperative group shows a relationship between muscle function and inflammatory parameters, with handgrip strength negatively correlated with hs-CRP and leptin levels. Inflammation is linked to obesity and several chronic complications, including insulin resistance and type 2 diabetes [40,41]. The excessive production of pro-inflammatory cytokines causes damage to muscle fiber and protein content diameter, which can negatively impact the production of muscle strength [42]. In the postoperative group, we also found a relationship between leptin and parameters of muscle quality and functionality; leptin was negatively correlated with appendicular lean mass and handgrip strength. Elevated leptin levels impact muscle dysfunction by impairing lipid metabolism and intramuscular fatty acid oxidation [43,44]. In obese individuals, leptin receptors are expressed in muscle; however, the state of leptin resistance characteristic of obesity causes muscle atrophy and may worsen sarcopenic obesity [45]. Our results corroborate the findings of [46] who associated high levels of leptin with an increased risk for sarcopenic obesity.

On the other hand, in the preoperative group, adiponectin was positively correlated with gait speed in men and, in the postoperative group, was positively correlated with handgrip strength in men, which indicates that the improvement in the inflammatory response exerts a direct influence on muscle quality and functionality.

Sarcopenic obesity is mainly characterized by abdominal obesity. It is also associated with insulin resistance, causing metabolic damage to the muscle and presenting sarcopenia clinically [47]. Our study corroborates the finding of HOMA-IR being significantly higher in the preoperative group than in the postoperative group. In the preoperative group, HOMA-IR was negatively correlated with gait speed in men indicating the impact of insulin resistance on muscle function. The preoperative group showed higher values of glycated hemoglobin, characterizing them with reduced glucose tolerance. On the other hand, the postoperative group had a better lipid profile and liver profile compared to the preoperative group, results possibly resulting from weight loss.

As limitations of this study, the cross-sectional nature made it impossible for us to evaluate the same patients in the two periods studied. The small number of patients evaluated was another negative point. However, our study has some strengths, including the assessment, for the first time, of the phase angle and sarcopenia in the late postoperative period of Roux-en-Y bypass. Such assessments allow us to precisely evaluate the relationship between changes in body composition and bioimpedance components since it is known that a recent post-surgical evaluation may present bias concerning weight not stabilizing as a result of the surgical procedure. The other strong point is evaluating young patients since most studies provide us with data related to sarcopenia and phase angle in individuals with advanced age. However, the literature has already described sarcopenia and changes in phase angle with age well, regardless of changes in body composition.

In conclusion, our findings suggest that phase angle and other BIA components provide a good correlation with sarcopenia, especially regarding the reduction in muscle function, in an early manner. The BIA and handgrip strength tests are well-validated and low-cost tests. Such methods have excellent clinical practice applicability, allowing early intervention in obese and obese sarcopenic patients who will be submitted to gastric bypass, aiming to minimize functional limitations and metabolic complications from sarcopenia.

## Figures and Tables

**Table 1:** Characteristics of preoperative and postoperative groups.

Parameters	Preoperative				Postoperative			
	Women	Men	All	*p* Value	Women	Men	All	*p* Value
Number of participants	28	11	39	-	18	12	30	-
Age (years)	36.57 ± 6.75	41.82 ± 12.25	38.1 ± 8.81	0.9281 ^1^ 0.7435 ^a^	37.1 ± 5.58	42.17 ± 8.45	39.1 ± 7.19	0.9019 ^2^
Weight (kg)	117.81 ± 17.21	131.11 ± 19.91	121.6 ± 18.75	<0.0001 ^1,^* <0.0001 ^a,^*	69.39 ± 10.52	83.67 ± 11.93	75.1 ± 13.02	<0.0001 ^2,^*
Height (m)	1.62 ± 0.07	1.74 ± 0.06	1.66 ± 0.08	0.8745 ^1^ 0.7711 ^a^	1.62 ± 0.06	1.73 ± 0.06	1.70 ± 0.08	0.7808 ^2^
BMI (kg/m²)	44.68 ± 5.68	42.93 ± 4.94	44.2 ± 5.47	<0.0001 ^1,^* <0.0001 ^a,^*	26.53 ± 3.04	27.9 ± 4.11	27.1 ± 3.51	<0.0001 ^2,^*
WC (cm)	118.55 ± 9.99	129.95 ± 13.78	121.8 ± 12.16	<0.0001 ^1,^* <0.0001 ^a,^*	80.44 ± 9.27	91.75 ± 11.44	85 ± 11.48	0.0001 ^2,^*
NC (cm)	40.2 ± 2.43	47.65 ± 3.85	42.4 ± 4.46	<0.0001 ^1,^* <0.0001 ^a,^*	32.39 ± 2.07	37.67 ± 1.89	34.5 ± 3.28	<0.0001 ²^,^*
SBP (mmHg)	128.86 ± 14.54	138.36 ± 19.55	131.5 ± 16.42	0.0016 ^1,^** 0.0002 ^a,^**	113.89 ± 12.38	119.67 ± 13.40	116.2 ± 12.89	0.0244 ^2^ **
DBP (mmHg)	81.29 ± 18.10	89.82 ± 11.88	83.7 ± 16.88	0.0311 ^1,^** 0.0077 ^a,^**	75 ± 10.90	79.83 ± 12.25	76.5 ± 11.5	0.0601 ^2^
Postoperative time (months)	-	-	-	-	45.75 ± 10.47	44.5 ± 8.89	45.1 ± 9.5	-
Weight loss postoperative (kg)	-	-	-	-	56.64 ± 13.97	66.17 ± 50.90	61.0 ± 35.6	-
Weight loss postoperative (%)	-	-	-	-	55.27 ± 7.40	58.93 ± 10.98	57.0 ± 9.2	-

Values are expressed as mean ± standard deviation. Abbreviations: BMI (Body Mass Index); WC (Waist Circumference); NC (Neck Circumference); SBP (Systolic Blood Pressure); DBP (Diastolic Blood Pressure); FM (Fat Mass); FFM (Fat-Free Mass). * *p* < 0.0001 statistical significance; ** *p* <0.05 statistical significance. ^1^ Difference between groups for females; ^2^ Difference between groups for males; ^a^ Difference between pre- and postoperative groups. Mann–Whitney Test.

**Table 2:** Body composition and bioimpedance parameters in pre- and postoperative groups.

Parameters	Preoperative				Postoperative			
	Women	Men	All	*p* Value	Women	Men	All	*p* Value
Number of participants	28	11	39	-	18	12	30	-
FM (%)	46.29 ± 3.64	35.93 ± 3.13	53 ± 11.49	<0.0001 ^1,^* <0.0001 ^a,^*	30.48 ± 6.61	23.96 ± 7.32	21.3 ± 7.93	0.0012 ^2,^**
FM (kg)	55.27 ± 11.24	47.49 ± 10.63	69.1 ± 12.29	<0.0001 ^1,^* <0.0001 ^a,^*	21.63 ± 7.30	20.68 ± 9.10	53.9 ± 9.15	0.0002 ^2,^**
FFM (kg)	63.21 ± 7.06	83.58 ± 10.24	43.3 ± 5.89	<0.0001 ^1,^* <0.0001 ^a,^*	47.76 ± 4.9	62.98 ± 5.67	27.9 ± 7.51	0.0001 ^2,^*
PhA (degree)	4.44 ± 1.53	4.93 ± 0.84	4.6 ± 1.37	0.0036 ^1,^** <0.0007 ^a,^**	3.38 ± 0.92	4.10 ± 0.79	3.7 ± 0.93	0.0337 ^2,^**
Resistance (Ohm Ω)	497.56 ± 110.07	384.55 ± 50.15	464.8 ± 109	0.0199 ^1,^** 0.0026 ^a,^**	559.56 ± 61.92	470.50 ± 47.25	523.9 ± 71.2	0.0012 ^2,^**
Reactance (Ohm Ω)	39.07 ± 22.52	33.45 ± 8.66	37.4 ± 19.6	0.5465 ^1^ 0.6652 ^a^	33.28 ± 10.11	33.83 ± 7.66	33.5 ± 9.1	0.8532 ^2^
BMR (kcal /d)	1920.41 ± 215.95	2541.59 ± 311.52	2100.1 ± 374.7	<0.0001 ^1,^* <0.0001 ^a,^*	1452.17 ± 148.71	1915.17 ± 172.47	1637.4 ± 278.3	0.0002 ^2,^**

Values are expressed as mean ± standard deviation. Abbreviations: FM (Fat Mass); FFM (Fat-Free Mass); BMR (Basal Metabolic Rate); PhA (Phase Angle). * *p* < 0.0001 statistical significance; ** *p* < 0.05 statistical significance. ^1^ Difference between groups for females. ^2^ Difference between groups for males. ^a^ Difference between pre- and postoperative groups. Mann–Whitney Test.

**Table 3:** Classification criteria for sarcopenia between groups.

Parameters	Preoperative		Postoperative	
	Women	Men	Women	Men
Number of participants	28	11	18	12
Handgrip strength (<20 kgf; <30 kgf) % (n)	17.86% (5)	9.09 % (1)	22.22% (4)	16.66% (2)
Gait speed (<0.8 m/s) % (n)	3.57% (1)	¥	¥	¥
ALM (kg/h²) % (n)	¥	¥	¥	¥

Abbreviations: ALM (Appendicular Lean Mass). ¥: no participant for the variable. Chi-squared Test.

**Table 4:** Biochemical, metabolic, and inflammatory parameters in preoperative and postoperative groups.

Parameters	Preoperative				Postoperative			
	Women	Men	All	*p* Value	Women	Men	All	*p* Value
Number of participants	28	11	39	-	18	12	30	-
Insulin (mg/dL)	17.53 ± 8.58	20.52 ± 5.23	18.4 ± 7.8	<0.0001 ^1^ <0.0001 ^a,^*	4.23 ± 2.29	4.88 ± 2.08	4.5 ± 2.2	<0.0001 ^2,^**
Glucose (mg/dL)	88.20 ± 13.70	111.10 ± 56.10	94.8 ± 33.1	0.0003 ^1^ <0.0001 ^a,^*	77.33 ± 4.94	75.80 ± 5.45	76.7 ± 5.1	0.0002 ^2,^**
HOMA-IR	3.81 ± 2.40	5.47 ± 2.43	4.4 ± 2.4	<0.0001 ^1^ <0.0001 ^a,^*	0.81 ± 0.45	0.91 ± 0.39	0.8 ± 0.4	<0.0001 ^2,^*
HbGli (%)	5.69 ± 0.81	6.69 ± 2.12	6.0 ± 1.4	0.0627 ¹ 0.0003 ^a,^**	5.26 ± 0.40	5.13 ± 0.27	5.2 ± 0.4	0.0005 ^2,^**
Total Cholesterol (mg/dL)	174.04 ± 28.68	190.82 ± 35.17	179 ± 31.2	<0.0001 ^1^ <0.0001 ^a,^*	132.17 ± 25.52	146.00 ± 22.68	137.7 ± 25	0.0021 ^2,^**
LDL–c (mg/dL)	107.07 ± 26.06	122.00 ± 27.18	111.4 ± 26.9	0.0001 ^1^ <0.0001 ^a,^*	72.83 ± 25.13	80.25 ± 24.21	75.8 ± 24.6	0.0019 ^2,^**
HDL-c (mg/dL)	40.12 ± 7.20	36.55 ± 6.67	39.1 ± 7.1	0.0010 ^1^ <0.0001 ^a,^*	50.18 ± 10.10	54.08 ± 13.72	51.8 ± 11.7	0.0012 ^2,^**
VLDL-c (mg/dL)	26.70 ± 10.26	32.36 ± 8.55	28.3 ± 10	<0.0001 ^1^ <0.0001 ^a,^*	13.11 ± 4.16	32.36 ± 8.55	13.4 ± 3.9	<0.0001 ^2,^*
Triglyce-rides(mg/dL)	129.74 ± 47.40	165.18 ± 50.81	140 ± 50.4	<0.0001 ^1^ <0.0001 ^a,^*	65.56 ± 20.54	69.58 ± 18.28	67.2 ± 19.4	<0.0001 ^2,^*
ALT (mg/dL)	18.35 ± 7.54	32.67 ± 9.46	21.3 ± 9.8	0.0011 ^1,^** 0.0007 ^a,^**	12.00 ± 3.46	16.17 ± 4.17	13.7 ± 4.3	0.0031 ^2^
AST (mg/dL)	17.13 ± 3.45	25.17 ± 4.40	18.8 ± 4.9	0.1853 ^1^ 0.3023 ^a^	15.94 ± 4.05	20.50 ± 6.22	17.8 ± 5.5	0.1328 ^2^
Uric Acid (mg/dL)	7.30 ± 5.76	8.92 ± 5.08	7.6 ± 5.6	<0.0001 ^1^ <0.0001 ^a,^*	3.48 ± 0.89	4.91 ± 1.04	4.1 ± 1.2	0.0077 ^2^
GGT (mg/dL)	21.43 ± 11.02	40.52 ± 20.77	25.4 ± 15.3	<0.0001 ^1^ <0.0001 ^a,^*	9.07 ± 2.22	15.50 ± 5.54	11.9 ± 5.1	0.0274 ^2^
hs-CRP (mg/dL)	4.87 ± 1.38	4.68 ± 1.63	4.82 ± 1.44	<0.0001 ^1^ <0.0001 ^a,^*	0.94 ± 1.68	1.50 ± 1.48	1.16 ± 1.60	0.0012 ^2^
Leptin (ng/mL)	75.30 ± 29.22	41.87 ± 17.79	65.87 ± 30.37	<0.0001 ^1^ <0.0001 ^a^	19.50 ± 12.26	11.20 ± 8.28	16.18 ± 11.45	0.0004 ^2^

Values are expressed as mean ± standard deviation. Abbreviations: LDL-c (Low-Density Lipoprotein cholesterol); HDL-c (High-Density Lipoprotein cholesterol); VLDL-c (Very Low-Density Lipoprotein cholesterol); HOMA-IR (Homeostatic Model Assessment-Insulin Resistance); HbGli (Glycated Hemoglobin); ALT (Alanine Aminotransferase); GGT (Gamma Glutamyl Transpeptidase); hs-CRP (ultra-sensitive C-Reactive Protein). * *p* < 0.0001 statistical significance; ** *p* < 0.05 statistical significance. ^1^ Difference between groups for females; ^2^ Difference between groups for males; ^a^ Difference between pre- and postoperative groups. Mann–Whitney Test.

**Table 5:** Correlation between sarcopenia and parameters of bioimpedance, inflammation, and HOMA-IR in preoperative and postoperative groups.

Parameters	Reactance Coefficient *p* Value	Resistance Coefficient *p* Value	PhA Coefficient *p* Value	Hs- CRP Coefficient *p* Value	Leptin Coefficient *p* Value	HOMA-IR Coefficient *p* Value
Preoperative						
Handgrip strength	−0.21901 0.2062	−0.63669 <0.0001 *	0.18351 0.2913	−0.45436 0.0197 **	−0.46505 0.0043 **	0.32455 0.0611
Gait speed	−0.05695 0.7341	−0.36220 0.0254 **	0.12684 0.4480	0.24500 0.2089	−0.23532 0.1493	−0.09166 0.5895
ALM	−0.49485 0.0034 **	−0.65797 <0.0001 *	−0.05702 0.7526	−0.25562 0.2391	−0.15701 0.3752	0.34941 0.0500
Postoperative						
Handgrip strength	−0.00669 0.9720	−0.47147 0.0085 **	0.24538 0.1912	0.32571 0.1496	−0.40200 0.0277 **	0.16796 0.3750
Gait speed	−0.30006 0.1138	−0.32540 0.0850	−0.12580 0.5155	−0.05474 0.8137	0.07770 0.6887	0.20128 0.2951
ALM	−0.26003 0.2094	−0.72668 <0.0001 *	0.09385 0.6555	−0.03735 0.8908	−0.41000 0.00418 **	0.10692 0.6110

Abbreviations: ALM (Appendicular Lean Mass), PhA (Phase Angle); HOMA-IR (Homeostatic Model Assessment-Insulin Resistance); hs-CRP (ultra-sensitive C-Reactive Protein). * *p* < 0.0001 statistical significance; ** *p* < 0.05 statistical significance. Spearman’s linear correlation coefficient.

**Table 6:** Handgrip strength, gait speed, and appendicular lean mass in sarcopenic individuals between preoperative and postoperative groups.

Parameters	Preoperative	Postoperative	
	(N = 6)	(N = 6)	*p*-Value
Handgrip strength	19.4 ± 3.1	21.5 ± 4.7	0.3358
Gait speed (<0.8m/s)	1.1 ± 0.2	1.0 ± 0.2	0.9362
ALM (kg/h²)	9.9 ± 1.3	7.7 ± 1.2	0.0367 **

Values are expressed as mean ± standard deviation Abbreviations: ALM (Appendicular Lean Mass). ** *p* < 0.05 statistical significance. Mann–Whitney Test.

## Data Availability

The data presented in this study are available on request from the corresponding author.

## Appendix A

**Table A1:** Correlation between sarcopenia and parameters of bioimpedance, inflammation, and HOMA-IR in preoperative group stratified by sex.

Parameters	Reactance Coefficient *p* Value	Resistance Coefficient *p* Value	PhA Coefficient *p* Value	Adiponectin Coefficient *p* Value	HOMA-IR Coefficient *p* Value	IL1-β Coefficient *p* Value
Men						
Handgrip strength	−0.09133 0.7894	−0.17273 0.6115	0.02727 0.9366	0.21818 0.5192	0.01818 0.9577	0.83636 0.00013 **
Gait speed	0.22375 0.5084	0.42727 0.1899	0.07273 0.8317	0.66364 0.0260 **	−0.609 0.0467 **	0.23636 0.4841
ALM	−0.16247 0.6332	−0.44647 0.1686	0.03189 0.9258	−0.68793 0.0193 **	0.20957 0.5363	0.53759 0.0881
Women						
Handgrip strength	−0.29998 0.1544	−0.36894 0.0760	−0.02958 0.8909	−0.41654 0.0677	0.10568 0.6231	0.000308 0.9883
Gait speed	−0.09145 0.6501	−0.34574 0.0773	0.09881 0.6239	0.38246 0.0790	−0.08718 0.6654	−0.10012 0.6122
ALM	−0.64009 0.0013 **	−0.56538 0.006 **	−0.19938 0.3737	−0.12998 0.6190	0.20057 0.3708	0.09933 0.6520

Abbreviations: ALM (Appendicular Lean Mass), PhA (Phase Angle); HOMA-IR (Homeostatic Model Assessment-Insulin Resistance); hs-CRP (ultra-sensitive C-Reactive Protein); IL1-β (interleukin 1 beta). ** *p* < 0.05 statistical significance. Spearman’s linear correlation coefficient.

**Table A2:** Correlation between sarcopenia and parameters of bioimpedance, inflammation, and HOMA-IR in postoperative group stratified by sex.

Parameters	Reactance Coefficient *p* Value	Resistance Coefficient *p* Value	PhA Coefficient *p* Value	Hs-CRP Coefficient *p* Value	HOMA-IR Coefficient *p* Value	Adiponectin Coefficient *p* Value
Men						
Handgrip strength	−0.10858 0.7369	−0.20280 0.5273	0.03497 0.9141	−0.05406 0.9084	0.10490 0.7456	0.72727 0.0074 **
Gait speed	−0.15993 0.6195	0.41053 0.1850	0.01404 0.9655	0.65455 0.1106	0.09474 0.7696	0.42807 0.1651
ALM	−0.52254 0.2289	−0.78571 0.0362 **	−0.42857 0.3374	1.00000	0.39286 0.3833	0.67857 0.0938
Women						
Handgrip strength	−0.16762 0.5062	0.07537 0.7663	−0.25697 0.3033	0.06007 0.8384	−0.03818 0.8804	−0.05882 0.8167
Gait speed	−0.48030 0.0510 **	−0.11548 0.6590	−0.39411 0.1175	0.22409 0.4412	0.15838 0.5438	−0.14487 0.5790
ALM	−0.41180 0.0895	−0.41507 0.0867	−0.26316 0.2914	−0.10679 0.7163	−0.04025 0.8740	−0.12074 0.6332

Abbreviations: ALM (Appendicular Lean Mass), PhA (Phase Angle); HOMA-IR (Homeostatic Model Assessment-Insulin Resistance); hs-CRP (ultra-sensitive C-Reactive Protein) ** *p* < 0.05 statistical significance. Spearman’s linear correlation coefficient.
